# Neoadjuvant Intravesical Mitomycin C for NMIBC: A Phase III Single-Center, Open-Label Randomized Clinical Trial

**DOI:** 10.3390/cancers18091444

**Published:** 2026-04-30

**Authors:** Roberto Contieri, Alberto Saita, Marco Paciotti, Alessandro Uleri, Pier Paolo Avolio, Vittorio Fasulo, Ludovica Cella, Stefano Mancon, Federica Sordelli, Alessio Finocchiaro, Giuseppe Garofano, Paola Arena, Chiara Pozzi, Andrea Gatti, Michela Lizier, Miriam Cieri, Piergiuseppe Colombo, Nicolò Maria Buffi, Giovanni Lughezzani, Paolo Casale, Massimo Lazzeri, Rodolfo Hurle

**Affiliations:** 1 Department of Biomedical Sciences, Humanitas University, 20072 Pieve Emanuele, Italymarco.paciotti@humanitas.it (M.P.); alessandro.uleri@humanitas.it (A.U.); pierpaolo.avolio@humanitas.it (P.P.A.); vittorio.fasulo@humanitas.it (V.F.); ludovica.cella@humanitas.it (L.C.); stefano.mancon@humanitas.it (S.M.); federica.sordelli@humanitas.it (F.S.); alessio.finocchiaro@humanitas.it (A.F.); paola.arena@humanitas.it (P.A.); miriam.cieri@humanitas.it (M.C.); piergiuseppe.colombo@humanitas.it (P.C.); nicolo.buffi@hunimed.eu (N.M.B.); giovanni.lughezzani@hunimed.eu (G.L.); 2 Department of Urology, IRCCS Humanitas Research Hospital, 20089 Rozzano, Italy; alberto.saita@humanitas.it (A.S.); paolo.casale@humanitas.it (P.C.); rodolfo.hurle@humanitas.it (R.H.); 3 IRCCS Humanitas Research Hospital, Via Manzoni 56, 20089 Rozzano, Italy; chiara.pozzi@humanitas.it (C.P.); andrea.gatti@humanitas.it (A.G.); michela.lizier@humanitas.it (M.L.); 4 Department of Pathology, IRCCS Humanitas Research Hospital, 20089 Rozzano, Italy

**Keywords:** non-muscle invasive bladder cancer, neoadjuvant therapy, mitomycin C, recurrence-free survival, immunogenic cell death, intravesical chemotherapy

## Abstract

**Background and Objective:** Transurethral resection of bladder tumor (TURBT) is the standard for non-muscle-invasive bladder cancer (NMIBC), yet recurrence rates remain high. This study evaluates the safety, tolerability, and efficacy of neoadjuvant intravesical mitomycin C (neoMMC) before TURBT in reducing recurrence and improving surgical outcomes. **Methods:** This randomized phase III trial enrolled patients with primary or recurrent NMIBC. Participants were randomized 1:1 to a neoadjuvant group receiving two instillations of MMC (day −14 and −7) before TURBT, or a control group undergoing standard TURBT without neoadjuvant treatment. The primary endpoint was 12-month recurrence-free survival (RFS). Secondary endpoints included surgical quality (complete resection, cauterization only, absence of residual tumor) and safety. Exploratory endpoints included histopathologic response and time to recurrence. **Key Findings and Limitations:** Among 95 patients (48 neoMMC, 47 controls), baseline characteristics were balanced. After a median follow-up of 19.4 months, recurrences occurred in 9 StA and 4 NeoA patients, with one progression to MIBC in the NeoA arm. RFS did not differ significantly between groups at 12 or 18 months. Neoadjuvant MMC was well tolerated, with only grade 1–2 AEs. Exploratory microbiota analyses suggested that neoadjuvant MMC modulated urinary microbial diversity and was associated with a microbiota profile more similar to that observed in non-recurrent patients. Limitations include single-center design and relatively short follow-up. **Conclusions and Clinical Implications:** Neoadjuvant intravesical MMC before TURBT was feasible and well tolerated in patients with NMIBC, with no unexpected safety signals. In this prematurely terminated and underpowered trial, no significant improvement in RFS was observed. Larger adequately powered studies are needed to clarify the oncologic efficacy of this approach.

## Simple Summary

Bladder cancer that has not invaded the muscle layer is usually treated by removing the tumor through the bladder, but the disease often comes back. In this study, we tested whether giving two doses of mitomycin C directly into the bladder before surgery could make treatment more effective. We compared patients who received this pre-surgical treatment with patients who underwent standard surgery alone. Although recurrence-free survival at 12 and 18 months was not significantly different, the pre-surgical treatment was safe, well tolerated, and appeared to improve tumor response and the quality of tumor removal. The treatment was also associated with changes in the urinary microbiota, which may be linked to lower recurrence risk. These findings suggest that giving mitomycin C before surgery may be a promising additional strategy for selected patients with non-muscle-invasive bladder cancer and should be tested further in larger multicenter studies.

## 1. Introduction

The standard treatment for NMIBC consists of transurethral resection of bladder tumor (TURBT), often combined with adjuvant intravesical therapies to reduce recurrence and progression [1]. These therapies include either intravesical chemotherapy and immunotherapy. Moreover, a single immediate postoperative instillation of chemotherapy has demonstrated a 14% reduction in the 5-year recurrence rate, underscoring its importance in clinical practice in selected patients [2].

This effect may be attributed to the elimination of circulating tumor cells post-TURBT, as well as its ablative impact on residual tumor cells at the resection site and on undetected microscopic lesions. Among the available intravesical chemotherapy agents, mitomycin C (MMC) has shown consistent safety and efficacy across multiple clinical regimens. MMC is an antineoplastic antibiotic; its mechanism of action involves alkylation and cross-linking of DNA strands, primarily at guanine-cytosine-rich regions, leading to inhibition of DNA replication and transcription, which induces apoptosis in rapidly proliferating cancer cells.

Forty years after its introduction in clinical practice, MMC remains one of the most widely used drugs for both ablation and prophylaxis of recurrence of NMIBC [3,4]. Although adjuvant MMC has potential benefits, its use in clinical practice remains limited due to logistical challenges and safety concerns. This underscores the importance of evaluating its efficacy in the neoadjuvant setting to better define its role in NMIBC management. However, while MMC has already been investigated in the neoadjuvant setting with various protocols, these studies have primarily focused on its ablative effects [5,6,7]. The precise dose and schedule of intravesical chemotherapy required to effectively reduce the recurrence risk in NMIBC remain uncertain and warrant further investigation.

Our group previously identified a novel immune-related mechanism of action for MMC involving the induction of immunogenic cell death (ICD) [8]. ICD enhances anti-tumor immunity through the release of damage-associated molecular patterns, including HMGB1, which activate innate immune responses and tumor antigen presentation. However, the effectiveness of MMC-induced ICD seems to rely on the tumor cells’ ability to undergo metabolic reprogramming. Specifically, “responder” tumors demonstrate mitochondrial reprogramming and ICD induction, while “non-responder” tumors with high glycolytic activity and reduced expression of respiratory chain protein Complex I (termed “Mitomarker”) exhibit resistance to ICD [8].

We and others have reported that bladder cancer patients exhibit distinct urinary microbiota profiles compared to healthy individuals [8,9]. Some bacteria, such as *Fusobacterium*, have been identified as potentially protumorigenic, being more abundant in bladder cancer patients [10,11]. Interestingly, the composition of the urinary microbiome may influence the response to bladder cancer treatments, such as Bacillus Calmette-Guerin (BCG) therapy [12,13]. Finally, specific genera, such as *Herbaspirillum*, *Porphyrobacter* and *Bacteroides*, are enriched in patients with a high risk of recurrence, highlighting the potential of the urinary microbiome as a biomarker for disease progression and risk stratification [14]. We hypothesize here that anticipating MMC treatment may also modify the local microbiota composition towards one that is more conducive to treatment efficacy.

These findings support the rationale for investigating MMC in the neoadjuvant setting, where a larger tumor burden may enhance the activation of anti-cancer immune responses. Moreover, the early initiation of immune activation could potentially augment the efficacy of subsequent adjuvant intravesical therapies.

This Phase III, single-center, open-label randomized trial aims to evaluate the efficacy and safety of neoadjuvant MMC in NMIBC. By leveraging MMC’s immune-modulating properties and eventually microbiota composition, this study seeks to optimize NMIBC management and explore its potential role as neoadjuvant therapy.

## 2. Materials and Methods

### 2.1. Study Design and Duration

This open-label, single-center, prospective, randomized phase III clinical trial was conducted between January 2022 and May 2024. The primary objective was to evaluate the safety and efficacy of neoadjuvant intravesical MMC (Medac Pharma srl, Roma, Italy) in reducing recurrence rates in patients with NMIBC. The study was approved by Italian National Competent Authority (Italian Medicines Agency—AIFA) with the registration code: EudraCT-Number: 2021-003751-42, date: 8 July 2021. The study protocol was approved by the Institutional Review Board of the IRCCS Humanitas Research Hospital (Protocol ICH-013—UroNEOAd I, date: 25 January 2022) and adhered strictly to the principles outlined in the Declaration of Helsinki.

Eligible patients, identified based on predefined inclusion criteria, underwent screening. After obtaining informed consent, participants were randomly allocated to one of two groups: the control group or the neoadjuvant MMC (NeoA) group.

Variable block randomization was employed without stratification. As this was an open-label study, both physicians and patients were aware of the assigned treatment arm.

The enrollment period for the study, originally planned to span one year, was extended to two years due to unanticipated delays in patient recruitment. Despite the extension, the target sample size could not be reached. Consequently, the Principal Investigator and the Coordinating Physicians, decided to terminate the protocol prematurely and proceed with the analysis of the data collected to date.

### 2.2. Study Population and Inclusion Criteria

The study included adult patients (≥18 years) with a primary or recurrent diagnosis of bladder cancer who had not received prior adjuvant intravesical therapy. Clinical suspicion was based on hematuria or lower urinary tract symptoms and confirmed using imaging techniques, including ultrasound, CT, or MRI. Flexible cystoscopy with Narrow Band Imaging (NBI) was performed at the urologist’s discretion. Eligibility required at least one tumor ≥ 1 cm identified via imaging or cystoscopy, an Eastern Cooperative Oncology Group (ECOG) performance status of ≤2, a negative urine culture before therapy, and written informed consent in compliance with ICH/EU Good Clinical Practice guidelines.

### 2.3. Exclusion Criteria

Exclusion criteria included clinical evident muscle invasive bladder cancer (MIBC) or metastatic disease, hypersensitivity to MMC or its components, major non-diagnostic surgeries within the past three years, malignancies of the upper urinary tract or prostatic urethra, or other active cancers within the last three years. Patients with significant urologic abnormalities (e.g., urethral strictures or hypospadias) interfering with therapy, as well as pregnant or breastfeeding individuals, were excluded. Other exclusions included participation in therapeutic trials within the past four weeks, recent (<3 months) immunomodulatory therapy, active infections of the urinary tract, chronic inflammatory conditions (e.g., inflammatory bowel disease), or ongoing antibiotic use.

### 2.4. Study Interventions and Endpoints

Patients in the NeoA group underwent tumor biopsy on day −14 and received two intravesical MMC instillations, administered on days −14 and −7, prior to TURBT. The neoadjuvant MMC instillation schedule (at days −14 and −7 before TURBT) was selected to allow sufficient biological time for MMC-induced immunogenic cell death and immune activation to occur prior to tumor resection, based on preclinical and translational evidence supporting a time-dependent immune-mediated mechanism of action [8].

After TURBT, all patients with pathologically confirmed NMIBC, regardless of group allocation, received repeat TURBT (re-TURBT) and/or adjuvant treatment according to European Association of Urology (EAU) guidelines [1].

All patients received continuous postoperative saline irrigation for a minimum of 12 h, in accordance with the standard practice of the center.

Follow-up included a cystoscopy at 3 months, followed by risk-adapted surveillance schedules based on individual clinical risk groups, as outlined by the EAU guidelines.

The primary endpoint of the study was recurrence-free survival (RFS) at 12 months post-TURBT, defined as the proportion of patients without histologically confirmed recurrence within 12 months following TURBT. Secondary endpoints included the safety profile of neoadjuvant MMC administration and the progression-free survival (PFS). Progression was intended as the diagnosis of MIBC or distant metastasis during the follow up.

The severity of MMC related complications was assessed following the The National Cancer Institute Common Terminology Criteria for Adverse Events (NCI CTCAE) Version 4.03 [15].

An additional endpoint was the evaluation of the correlation between the histological findings from pre-instillation biopsies and those obtained from the TURBT.

Finally, a sensitivity analysis was conducted, including only patients who received adjuvant intravesical instillation (BCG or MMC).

### 2.5. DNA Isolation and Microbiome Analysis Through 16S rRNA Amplicon Sequencing

Catheterized urine samples from bladder cancer patients were collected at multiple time points: during neoadjuvant intravesical MMC treatment (NeoA arm), at TURBT, and at the 3-month follow-up (both StA and NeoA arms). Samples were immediately placed on dry ice and stored at −80 °C until DNA extraction. Bacterial DNA will be extracted using DNeasy PowerSoil Pro Kits (Qiagen, Hilden, Germany) according to the manufacturer’s procedures. DNA concentration will be measured using the NanoDrop spectrophotometer (Thermo Fisher Scientific, Waltham, MA, USA). 16S rRNA from V3V4 regions was amplified used specific primer sequences. All PCR reactions were carried out with 15 µL of Phusion High—Fidelity PCR Master Mix; 0.2 µM of primers, and about 10 ng template DNA. Sequencing libraries were generated and indexes were added. The library was checked with Qubit and real-time PCR for quantification and bioanalyzer for size distribution detection. Quantified libraries were pooled and sequenced on Illumina platform NovaSeq 6000 PE 2 × 250 bp, according to effective library concentration and data amount required. The bioinformatics pipeline included FLASH (v1.2.11) for merging paired-end reads and fastp (v0.23.1) for quality control of the merged sequences. Chimera removal was conducted using the vsearch package (v2.16.0) against the SILVA reference database and the following denoising was performed using the DADA2 algorithm within the QIIME 2 framework (v2022.2). Downstream analyses were carried out in R (v4.3.2), utilizing the vegan (v2.6-8) and MaAsLin2 (v1.16.0) packages. Diversity analyses included the calculation of several α-diversity metrics, namely Shannon and Simpson indices, as well as bacterial richness, while β-diversity was assessed using Bray–Curtis dissimilarity and PERMANOVA testing (n = 999). Generalized linear models (GLMs) were applied to evaluate α-diversity statistical significance, correcting for two amplicon sequence variants (ASVs) identified as PCR artifacts that were retained during the quality control. Multivariable associations between bacterial features and clinical variables were identified using the MaAsLin2 package, through the fitting of linear models (LMs) while adjusting for the aforementioned ASVs.

### 2.6. Statistical Analysis and Power Calculation

The primary aim of the study was to assess an improvement in 12-month relapse-free survival (RFS), corresponding to a hazard ratio (HR) of 0.6. Based on an assumed 12-month RFS rate of 70% in the control group and a recruitment period of 12 months, the required sample size was determined. With 80 patients per group and a significance level of α = 0.05, the study was calculated to have 80% power.

Categorical variables were presented as frequencies and percentages, while continuous variables were expressed as medians and interquartile ranges (IQR). Categorical variables were compared using Pearson’s χ^2^ test or Fisher’s exact test, while the Wilcoxon rank-sum test was used to test continuous variables.

The relationship between treatment arm and recurrence-free survival (RFS) was assessed using univariable Cox regression analysis. Kaplan–Meier curves were generated to illustrate RFS. Follow-up duration was calculated from the date of TURBT to the last recorded follow-up visit. Only participants with histologically confirmed NMIBC were included in the per protocol analyses. All statistical tests were two-sided, with a significance threshold set at *p* < 0.05. The Benjamini–Hochberg procedure for FDR in microbiome analysis was applied. Associations were considered significant when model Q < 0.25.

## 3. Results

**Figure 1:**
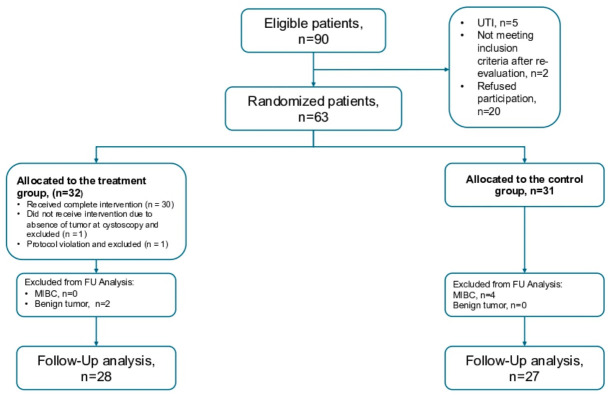
Flowchart of the randomized patients. UTI: Urinary Tract Infection, MIBC: Muscle-Invasive Bladder Cancer, FU: Follow-Up.

A total of 90 patients met the inclusion criteria and were screened preoperatively before undergoing TURBT. The study design and patient flow are illustrated in Figure 1.

Following the screening process, 63 patients were deemed eligible and randomized into two groups: 31 patients (49%) were allocated to the standard (StA) group, while 32 patients (51%) were assigned to the neoadjuvant (NeoA) group. During the biopsy phase, one patient in the NeoA group was found to have no lesions consistent with bladder cancer, leading to exclusion from the study. Another patient in the NeoA group was excluded because of protocol violation.

Twenty-nine patients (97%) in the neoadjuvant group underwent tumor biopsy on day −14, and all patients received the scheduled intravesical MMC instillations on days −14 and −7.

The two groups were well balanced in terms of baseline characteristics, ensuring comparability in the analysis (Table 1). The results of biopsy and the effect of MMC in the neoadjuvant cohort are presented in Table 2.

After excluding patients with absence of tumor or muscle-invasive bladder cancer (MIBC), 55 patients (27 in the StA arm and 28 in the NeoA arm) proceeded with protocol-defined follow-up.

After a median follow-up of 19.4 months, recurrence was observed in 9 patients in the StA group and 4 patients in the NeoA group. Only one patient from the NeoA group progressed to MIBC. This patient had T1 NMIBC and received adjuvant BCG, which was discontinued after the induction phase due to intolerance. Progression to MIBC occurred 15 months after the primary TURBT.

**Figure 2:**
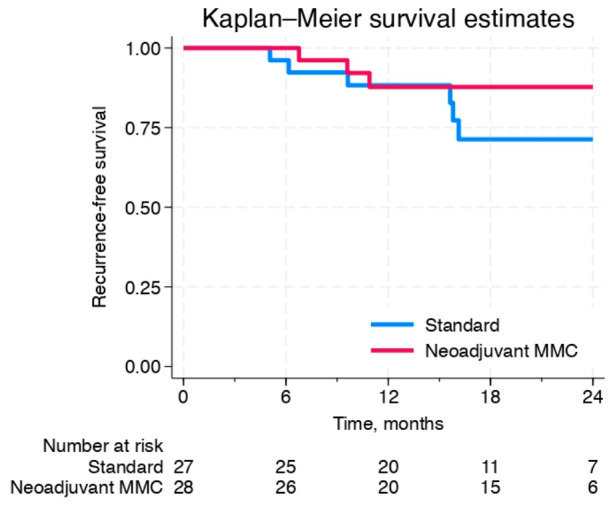
Kaplan–Meier estimates of recurrence-free survival for both treatment arms. At 12 months, the hazard ratio (HR) was 0.97 (95% CI: 0.2–4.8, *p* = 0.97), indicating that the primary endpoint was not met. However, an improvement in recurrence-free survival was observed at 18 months in the NeoA group (HR: 0.4; 95% CI: 0.1–1.8, *p* = 0.27).

Kaplan–Meier curves for RFS for both treatment arms are presented in Figure 2. At 12 months, the recurrence-free survival (RFS) was 88% (95% CI: 68–96) for the StA group and 88% (95% CI: 67–96) for the NeoA group, with a hazard ratio (HR) of 0.97 (95% CI: 0.2–4.8, *p* = 0.97). At 18 months, the RFS was 71% (95% CI: 46–86) for the StA group and 88% (95% CI: 67–96) for the NeoA group, corresponding to an HR of 0.4 (95% CI: 0.1–1.8, *p* = 0.27).

**Figure 3:**
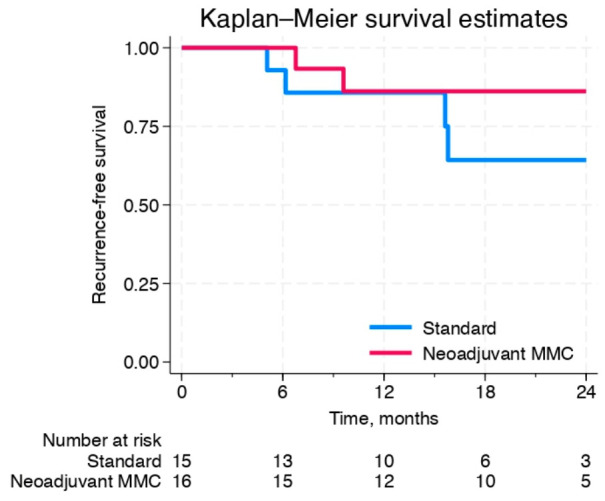
Kaplan–Meier estimates of recurrence-free survival for both treatment arms including only patients who received intravesical instillations (BCG or MMC).

In a sensitivity analysis including only patients who received adjuvant treatment (MMC or BCG) the 12-month RFS was 86% (95% CI: 54–96) for the StA group and 86% (95% CI: 55–96) for the NeoA group, with a HR of 0.86 (95% CI: 0.1–6.1, *p* = 0.9) (Figure 3). By 18 months, the RFS was 64% (95% CI: 29–85) in the StA group compared to 86% (95% CI: 55–96) in the NeoA group, with an HR of 0.4 (95% CI: 0.1–2.1, *p* = 0.27).

A total of five patients (14%) in the NeoA group experienced adverse events (AEs), amounting to eight reported events. The most common AE was hematuria (n = 3). All AEs were classified as grade 1 or 2, with no grade 3–5 events or systemic adverse events observed (Table 3). All reported adverse events in the NeoA group occurred after intravesical MMC instillations and prior to TURBT.

The comparison of histological grading between biopsy and TURBT showed confirmed WHO 2004/2020 and WHO 1973 grades in 67% and 64% of cases, respectively. Upgrading was identified in 20% and 23% of cases for WHO 2004/2020 and WHO 1973 grading systems, respectively, with no cases of downgrading. In three cases, biopsy specimens were inadequate for diagnostic evaluation due to insufficient tissue sampling, and in one case, the biopsy was not performed due to a protocol deviation.

**Figure 4:**
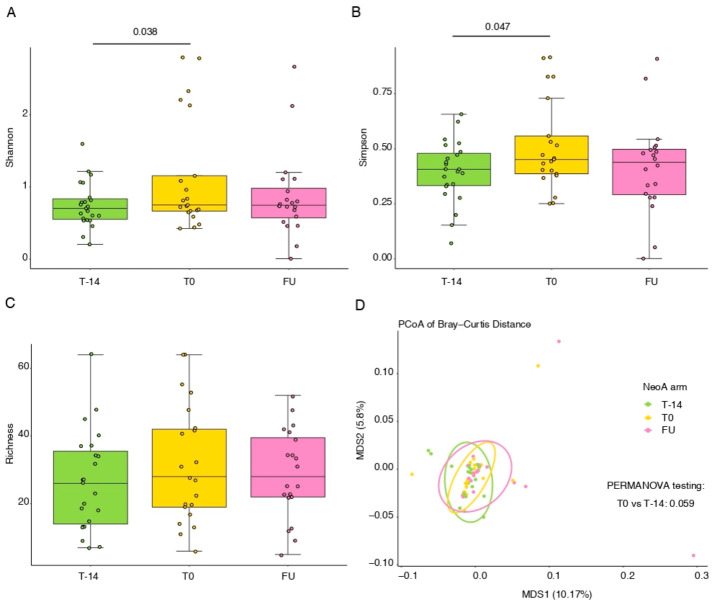
Alpha and Beta diversity analysis of the urinary microbiome in NeoA arm patients at different time points. (**A**), Shannon index; (**B**), Simpson index; (**C**), Observed genera (Bacterial Richness). Statistical significance was assessed using a generalized linear model (GLM). (**D**), Principal coordinate analysis (PCoA) of Bray–Curtis distance of catheter-collected urine samples. Statistical significance was evaluated using the PERMANOVA test. T-14, n = 23; T0, n = 21; FU. n = 20.

Urinary microbiota composition was assessed at different time points in the NeoA and StA groups using alpha and beta diversity indices. Samples were collected by sterile catheterization during clinical procedures, minimizing contamination from external urogenital microbiota. In the StA group, no significant differences in alpha or beta diversity were observed between TURBT (T0) and FU (Supplementary Figure S1). In contrast, the NeoA group showed a significant increase in microbial diversity at TURBT (T0) compared with the pre-treatment time point (T-14), as reflected by higher Shannon and Simpson indices, while bacterial richness (Observed Species Index) remained unchanged (Figure 4A–C). Although beta diversity did not show statistically significant changes over time, a trend toward separation between T-14 and T0 was observed (*p* = 0.059; Figure 4D). No differentially abundant genera were identified by MaAsLin2 between time points in either group.

**Figure 5:**
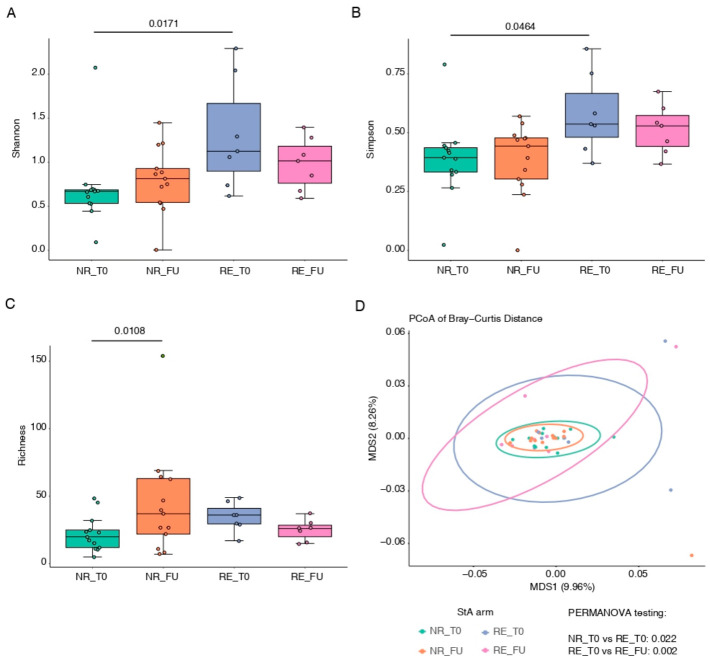
Alpha and Beta diversity analysis of the urinary microbiome in StA arm patients with (RE) or without (NR) recurrence at different time points. (**A**), Shannon index; (**B**), Simpson index; (**C**), Observed genera (Bacterial Richness). Statistical significance was assessed using a generalized linear model (GLM). (**D**), Principal coordinate analysis (PCoA) of Bray–Curtis distance of catheter-collected urine samples. Statistical significance was evaluated using the PERMANOVA test. NR_T0, n = 13; NR_FU, n = 13; RE_T0 n = 7.; RE_FU, n = 7.

Within the StA group, patients who experienced recurrence (RE) had significantly higher Shannon (*p* = 0.0171) and Simpson (*p* = 0.0464) diversity indices at T0 compared with NR patients (Figure 5A,B), with no differences in bacterial richness (Figure 5C). Beta diversity analysis (Bray–Curtis distances, PERMANOVA) demonstrated a significant difference in microbial composition between RE and NR patients at T0 (*p* = 0.022; Figure 5D). MaAsLin2 analysis identified several genera enriched in RE patients at T0, most of which decreased at FU, except for Varibaculum and Mobiluncus (Supplementary Figure S2A). Among these, Fusobacterium has been previously identified as potentially protumorigenic in bladder cancer [10,11], while Varibaculum and Actinomyces have been reported as more abundant in patients with urinary tract tumors compared with healthy individuals [16,17]. Actinotignum, although not directly linked to cancer, belongs to the same family as Actinomyces. Additional genera identified in RE patients have been implicated in other malignancies, including Campylobacter in colorectal cancer [18], Mobiluncus in endometrial cancer [19], and Finegoldia in cervical cancer and cervical intraepithelial neoplasia [20].

Among NR StA patients, bacterial richness was significantly higher at FU compared with T0 (*p* = 0.011; Figure 5C), with increased abundance of Ligilactobacillus, Klebsiella, Lactobacillus, Paracoccus, Escherichia–Shigella, and Cutibacterium (Supplementary Figure S2B). Conversely, RE patients exhibited a decrease in bacterial richness at FU (*p* = 0.007), although this did not remain significant after correction for multiple testing; however, beta diversity still showed a significant shift over time (*p* = 0.002; Figure 5C,D). No differentially abundant genera were detected between T0 and FU in RE patients.

**Figure 6:**
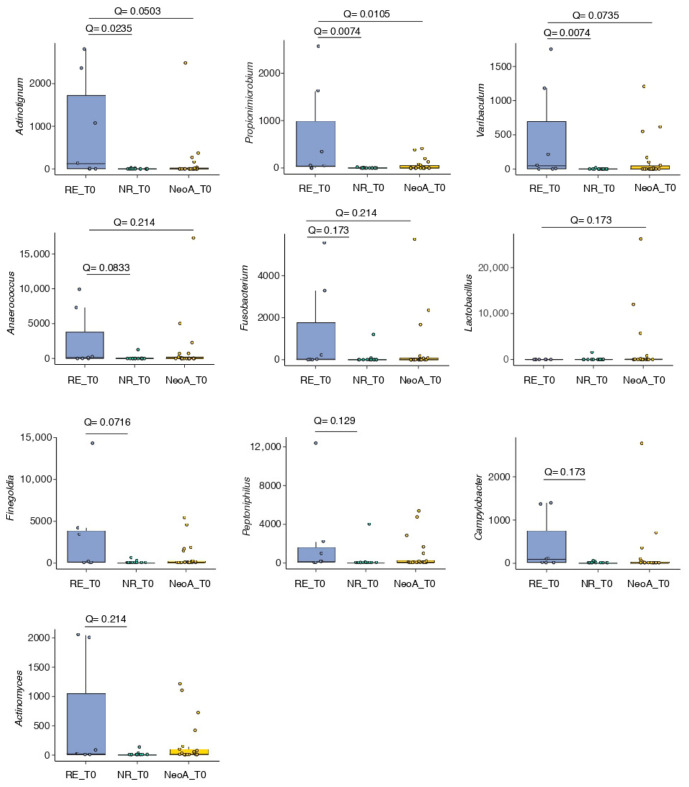
Differentially abundant genera in the urinary microbiome of StA arm patients with (RE) or without (NR) recurrence versus NeoA at T0. Genera significantly more abundant in RE patients at T0 versus NR patients at the same time point and Neo A patients at T0 (after 14 days MMC). Differential abundance analysis was performed using the MaAsLin2 package. The Benjamini–Hochberg procedure was applied to control the false discovery rate (FDR), and associations were considered significant at Q < 0.25. NR_T0, n = 13; RE_T0 n = 7; NeoA_T0, n = 21.

Comparative analyses at T0 showed that the NeoA microbiota was more similar to that of NR StA patients. While overall microbial composition did not differ significantly between NeoA and RE StA patients, MaAsLin2 analysis identified five genera (Propionimicrobium, Actinotignum, Varibaculum, Anaerococcus, Fusobacterium) that were significantly more abundant in RE StA patients than in either NR or NeoA patients, whereas Lactobacillus was enriched in the NeoA group (Figure 6). No differentially abundant genera were observed between NeoA and NR StA patients (Figure 6). The differences between NR and RE StA patients were consistent with those shown in Supplementary Figure S2, with Peptoniphilus replacing Mobiluncus. Overall, these findings suggest that neoadjuvant MMC may be associated with microbiota patterns resembling those observed in patients without recurrence, although these observations remain exploratory.

## 4. Discussion

In this open-label, single-center, prospective, randomized phase III clinical trial, we evaluated the safety and efficacy of two neoadjuvant MMC instillations administered 14 and 7 days before TURBT in patients with NMIBC. Although the study was terminated before reaching the target sample size, several findings warrant discussion.

Firstly, no statistically significant improvement in RFS was observed at 12 months in patients receiving two neoadjuvant MMC instillations compared with the control group. At 18 months, RFS estimates numerically favored the neoadjuvant arm; however, this difference did not reach statistical significance and should be interpreted with caution, given the limited sample size and follow-up duration. These results may suggest a potential delayed benefit of the neoadjuvant approach even if the statistical significance was not reached.

However, given the substantial shortfall from the planned sample size, all efficacy analyses should be regarded as exploratory and hypothesis-generating, and the non-significant result for the primary endpoint should not be interpreted as evidence of the absence of effect, but rather as an inconclusive finding in the context of a marked risk of type II error.

Our group previously demonstrated that MMC, when administered on a short schedule, induces ICD in tumor cells [8]. This mechanism may underlie the potential long-term recurrence reduction observed with neoadjuvant MMC. Furthermore, the benefit in RFS appears more pronounced among patients who received adjuvant treatment following TURBT (Figure 3). These findings support the hypothesis that MMC-induced ICD could enhance the efficacy of subsequent therapies, such as BCG, by priming the immune response.

Recently, two RCTs have reported contrasting results on the efficacy of neoadjuvant MMC administration in enhancing outcomes compared to the standard approach.

The interim analysis of the PRECAVE clinical trial reported no significant difference in RFS between patients receiving single immediate neoadjuvant MMC instillation and those in the control group overall. However, a substantial 80% reduction in the risk of early recurrences was observed in the subgroup of patients who did not receive adjuvant therapy [21]. Additionally, the protocol demonstrated high feasibility and safety, with minimal adverse events and excellent compliance; only 4 patients did not receive the planned neoadjuvant MMC instillation [21].

More recently, Lee et al. reported a significant reduction in RFS at 12 months in the intervention group (97% vs. 89%). However, the treatment protocol in that study involved MMC instillations administered 1 day and 4 h before TURBT [17].

The main characteristics and oncologic outcomes of these studies, together with those of the current study, are summarized in Table 4.

Secondly, neoadjuvant MMC demonstrated a favorable safety profile, with only five patients (14%) reporting AEs, all of which were grade 1–2. Our results align with those of Lee et al., who reported a 15% incidence of drug-related AEs in the intervention group [22]. It is important to acknowledge that MMC carries potential toxicities associated with extravasation and systemic absorption, as it is a vesicant chemotherapeutic agent with deep-tissue penetration and necrotizing properties [23]. Consequently, the EAU guidelines recommend omitting a single immediate instillation of chemotherapy in cases of evident or suspected bladder perforation or bleeding requiring bladder irrigation, leading to exclusion in a significant number of patients [1].

Notably, the neoadjuvant setting eludes these restrictions. In our study, all patients in the treatment arm were able to receive MMC instillations, highlighting the potential advantages of this approach in selected populations.

Thirdly, we did not observe a significant ablative effect of MMC on tumors; only two cases demonstrated a reduction in tumor size. Notably, the two cases with benign lesions did not exhibit any size reduction. This outcome is not entirely unexpected, as the study design and MMC administration regimen were not specifically optimized for tumor ablation.

The chemoablative role of MMC has been well-documented, with several studies comparing chemoresection with MMC to TURBT [3,5,6,7]. The intensive neoadjuvant MMC regimen (three instillations per week for two weeks) has shown to be the most effective achieving a higher complete tumor response rate (70.4%) compared to the weekly regimen (44.4%) with a similar safety profile [24].

Additionally, it is not surprising that two patients presented with benign lesions at TURBT, as benign lesions can often mimic papillary tumors. In fact, up to 20% of benign lesions may be mistaken for low-grade tumor recurrences due to their similar appearance [25].

Fourth, we reported a concordance rate of 67% and 64% for WHO 2004/2020 and WHO 1973 grading systems, respectively, in the comparison of histological grading between biopsy and TURBT; these findings highlight the diagnostic utility of pre-instillation biopsies but also underscore the importance of accurate sampling techniques.

Lastly, we observed that the urinary microbiota may be associated with recurrence in bladder cancer patients and that neoadjuvant MMC increased the diversity of the microbiota and reduced the abundance of species associated with recurrence. Thus, neoadjuvant treatment with MMC may support an immune-related activity but also may act as modulator of the tumor microenvironment through microbial reshaping. Further studies with larger cohorts are needed to validate these findings and to explore the therapeutic potential of microbiota modulation in bladder cancer management.

Although development of a microbial risk score would be an attractive translational objective, we did not pursue this analysis in the present study because the dataset was not designed for predictive model derivation and the limited number of recurrence events would have exposed any multivariable microbiome-based classifier to a high risk of overfitting. Current evidence nonetheless supports the potential relevance of this field: microbiome-based urinary classifiers have shown encouraging, albeit still preliminary, discriminatory performance in urothelial carcinoma, with an XGBoost-based model reporting an AUC of 0.927 in the training cohort and 0.811 in external validation [26]. These findings suggest that microbiome-informed risk stratification may be feasible, but robust score development will require larger prospective multicenter cohorts, standardized sampling and analytical workflows, and independent external validation before clinical implementation.

Despite its strengths, this study has several important limitations that should be considered when interpreting the findings. First, the target sample size was not reached because of slow accrual, and the study was therefore prematurely terminated. As a consequence, the trial was substantially underpowered for its primary endpoint, and all efficacy analyses should be regarded as exploratory and hypothesis-generating. In this context, the non-significant result for the primary endpoint cannot be interpreted as evidence of absence of effect, but rather as an inconclusive finding with a substantial risk of type II error. Moreover, the reduced sample size and early closure increase the likelihood of imprecise and unstable effect estimates, as well as chance baseline imbalances despite randomization. Second, the open-label, single-center design may have introduced selection, performance, and ascertainment bias, while post-randomization exclusions and the per-protocol efficacy analysis may have further increased the risk of attrition bias. These limitations were only partially mitigated by prospective randomized allocation, the overall balance of measured baseline characteristics between study arms, protocol-defined management and follow-up, and the use of objectively defined, histologically confirmed recurrence and progression endpoints. Third, patients in the control group did not receive immediate postoperative chemotherapy, as recommended by current guidelines; although all patients in both arms received continuous postoperative bladder irrigation, which may provide a recurrence-preventive effect comparable to immediate postoperative chemotherapy, this aspect should still be considered a limitation of the study design. Finally, the microbiome analyses were conducted in a small exploratory cohort, limiting the ability to draw firm conclusions regarding causal associations between urinary microbiota composition, MMC exposure, and oncologic outcomes. In addition, although 16S rRNA gene sequencing enables detection of low-abundance bacteria, it lacks species-level resolution and does not capture non-bacterial components such as fungi or viruses.

Despite its strengths, this study is not without limitations. Firstly, the target sample size was not achieved due to challenges in patient recruitment, which may have affected the statistical power and generalizability of the results. Consequently, the failure to achieve the primary endpoint may be attributable to the study being underpowered. Given that the planned sample size was not reached, it is possible that, with adequate statistical power, a significant difference in RFS might have been achieved. Additionally, given the substantially underpowered sample size, the non-significant p-value observed for the primary endpoint (12-month RFS) should be interpreted with caution, as it cannot reliably distinguish between a true lack of treatment effect and a Type II error.

This limitation necessitates caution when interpreting the findings, particularly regarding recurrence-free survival and safety outcomes. Understanding the reasons behind lower-than-expected patient enrollment is complex. A potential explanation is that some patients who declined participation were reluctant or unable to attend additional hospital visits beyond standard care. As a national referral center, our institution treats patients from various regions, many of whom face significant travel burdens. It is therefore plausible that the requirement to visit our center 14 and 7 days before the procedure for experimental treatment (neoadjuvant MMC instillations) deterred participation due to logistical challenges.

Beyond limited statistical power, the present study is also subject to potential design-related biases. The open-label, single-center design may have introduced selection, performance, and ascertainment bias, while the reduced sample size and premature study closure increase the risk of imprecise effect estimates and chance baseline imbalances despite randomization. In addition, post-randomization exclusions and the per-protocol efficacy analysis may have introduced attrition bias. These limitations were only partially mitigated by prospective randomized allocation, the overall balance of measured baseline characteristics between study arms, and protocol-defined management and follow-up. Nevertheless, residual bias cannot be excluded, and the findings should be interpreted with appropriate caution.

Secondly, the open-label design, while practical, introduces potential biases, including observer and selection bias.

Furthermore, patients in the control group were not scheduled to receive an immediate postoperative chemotherapy instillation, as recommended by current guidelines [1]. This should be considered a limitation of the study. However, all patients in both arms received continuous postoperative bladder irrigation, an approach that has been reported to provide a recurrence-preventive effect comparable to immediate postoperative chemotherapy [27,28].

The small sample size limits the ability to draw definitive conclusions or establish causal relationships between the urinary microbiome and bladder cancer recurrence as well as a direct relationship between MMC and microbiota composition. Finally, while 16S rRNA gene sequencing allows for the detection of low-abundance bacteria, it lacks species-level resolution and cannot detect non-bacterial organisms such as fungi or viruses.

## 5. Conclusions

This phase III randomized trial demonstrates that neoadjuvant intravesical MMC administered prior to TURBT is feasible and well tolerated in patients with NMIBC, with no unexpected safety signals. Within the limits of a prematurely terminated and underpowered study, no statistically significant improvement in recurrence-free survival was observed at either 12 or 18 months. Exploratory analyses of the urinary microbiota suggested potential MMC-associated changes; however, these findings are hypothesis-generating and should be interpreted with caution given their exploratory nature and the lack of confirmatory statistical significance.

Larger, adequately powered, multicenter trials with longer follow-up are required to definitively assess the oncologic efficacy of neoadjuvant MMC and to clarify whether modulation of the urinary microbiota has any clinically meaningful role in NMIBC outcomes.

## Figures and Tables

**Table 1:** Baseline patient characteristics stratified by treatment group. Treatment groups were well-balanced, with no statistically significant differences.

Variables	Control (n = 31)	Neoadjuvant MMC (n = 30)	
Age at surgery, median (IQR), years	65 (58, 75)	68 (59, 75)	0.8
Sex, n (%)			
Male	28 (90%)	26 (87%)	0.7
Primary tumor, n (%)			
Yes	29 (94%)	30 (100%)	0.2
Grade WHO 2004/2022, n (%)			
Low-Grade	17 (55%)	15 (50%)	0.3
High-Grade	14 (45%)	13 (43%)	
Benign	0 (0%)	2 (6.7%)	
Grade WHO 1973, n (%)			
G1	16 (52%)	15 (50%)	0.5
G2	3 (10%)	3 (10%)	
G3	12 (39%)	10 (33%)	
Benign	0 (0%)	2 (6.7%)	
Clinical T Stage, n (%)			
Benign	0 (0%)	2 (6.7%)	0.13
Ta	23 (74%)	24 (80%)	
CIS	0 (0%)	1 (3.3%)	
T1	4 (13%)	3 (10%)	
T2	4 (13%)	0 (0%)	
DM in resection, n (%)			
Yes	27 (87%)	26 (87%)	1
Presence of Urothelial subtypes, n (%)			
Yes	5 (16%)	2 (6.7%)	0.2
Presence of CIS, n (%)			
Yes	3 (10%)	1 (3.3%)	0.3
Tumor dimension, n (%)			
>3 cm	11 (35%)	11 (37%)	0.9
Multifocal tumor, n (%)			
Yes	11 (35%)	9 (30%)	0.6
Re-TURBT performed, n (%)			
Yes	6 (19%)	3 (10%)	0.3
Adjuvant intravesical therapy, n (%)			
None	15 (48%)	14 (47%)	0.8
BCG	9 (29%)	10 (33%)	
MMC	6 (19%)	6 (20%)	
Candidate for Early Instillation according to EAU *, n (%)			
No	16 (52%)	18 (60%)	0.5
Yes	15 (48%)	12 (40%)	

IQR: Interquartile range, CIS: Carcinoma in situ, EAU: European Association of Urology, WHO: World Health Organization, TURBT: Transurethral resection, BCG: Bacillus Calmette-Guerin, MMC: mitomycin C * Include patients with tumors classified as low-risk or those presenting with small papillary recurrences, presumed to be Ta LG/G1, occurring more than one year after the previous TUR.

**Table 2:** Results of biopsy and MMC effect in the neoadjuvant cohort. All patients underwent tumor biopsy and received intravesical MMC instillation on day −14, followed by a second MMC instillation on day −7, prior to undergoing transurethral resection (TURBT).

Variables	Neoadjuvant MMC (n = 30)
Grade WHO 2004/2020 at pre-MMC biopsy, n (%)	
Low-Grade	20 (67)
High-grade	6 (20)
Non evaluable *	4 (13)
Grade WHO 1973 at pre-mmc biopsy, n (%)	
G1	19 (63)
G2	3 (10)
G3	4 (13.5)
Non evaluable *	4 (13.5)
Comparison of WHO 2004/2020 Grade between Biopsy and TURBT	
Confirmed grade	20 (67)
Upgrading at TURBT	6 (20)
Downgrading at TURBT	0 (0)
Non evaluable at Biopsy *	4 (13)
Comparison of WHO 1973 Grade between Biopsy and TUR	
Confirmed grade	19 (64)
Upgrading at TURBT	7 (23)
Downgrading at TURBT	0 (0)
Non evaluable at Biopsy *	4 (13)
Visual evaluation of mmc direct effect on tumor dimension, n (%)	
None	28 (93)
Tumor size reduction	2 (7)
Tumor ablation	0 (0)

WHO: World Health Organization, TURBT: Transurethral resection, MMC: mitomycin C. * In three cases, the biopsy specimens were inadequate for diagnostic evaluation due to insufficient tissue sampling. In one case, the biopsy was not performed because of a protocol deviation.

**Table 3:** Adverse events reported among patients who received neoadjuvant intravesical MMC instillation. Three patients experienced two adverse events each, while two patients experienced a single adverse event.

Adverse Events	Grade 1–2 (n = 30)
Hematuria, n (%)	3 (10%)
Dysuria, n (%)	2 (6.7)
Itchy scalp, n (%)	2 (6.7)
Skin erythema, n (%)	1 (3.3)

**Table 4:** Main characteristics and oncologic outcomes of studies evaluating neoadjuvant intravesical MMC before TURBT.

Study	Study Design	Intervention	MMC Schedule	n Control Group	n Intervention Group	Mitomycin C Dose (mg)	T Stage (All Patients, Control + Study)	Median Follow Up	Recurrence Rate	Progression Rate
Carrion et al. 2021 [21]	RCT	TURBT alone vs. neoadjuvant intravesical MMC before TURBT	1 intravesical MMC instillation after induction of anesthesia for 15 min before TURBT	60	64	40	Ta: 81% T1: 22%	13.2 mo (control); 18.3 mo (study)	Control: 11/60 (18.3%); Intervention: 8/64 (12.5%)	Control: 5/60 (8.3%); Intervention: 3/64 (4.7%)
Lee et al. 2022 [22]	RCT	TURBT alone vs. neoadjuvant intravesical MMC before TURBT	2 intravesical MMC instillation, on day −1 and 4 h before TURBT	38	33	40	Ta: 41% CIS: 4% T1: 55%	36.1 mo (control); 26.1 mo (study)	Control: 8/38 (21%); Intervention: 3/33 (9%)	Control: 3/38 (7.8%); Intervention: 0/33 (0%)
Current study	RCT	Standard approach vs. neoadjuvant intravesical MMC before TURBT	intravesical MMC instillations on day −14 and day −7 before TURBT	27	28	40	Ta: 85% CIS: 2% T1: 13%	19.4 mo	Control: 9/27 (33.3%); Intervention: 4/28 (14.3%)	Control: 0/27 (0%); Intervention: 1/28 (3.6%)

MMC, mitomycin C; TURBT, transurethral resection of bladder tumor; RCT, randomized controlled trial; CIS, carcinoma in situ; mo, months; h, hours.

## Data Availability

Full data is unavailable due to privacy restrictions.

## Supplementary Materials

The following supporting information can be downloaded at: https://www.mdpi.com/article/10.3390/cancers18091444/s1, Figure S1: Alpha and Beta diversity analysis of the urinary microbiome in StA arm patients at T0 and at follow up (FU). A, Shannon index; B, Simpson index; C, Observed genera (Bacterial Richness). Statistical significance was assessed using a generalized linear model (GLM). D, Principal coordinate analysis (PCoA) of Bray–Curtis distance of catheter-collected urine samples. Statistical significance was evaluated using the PERMANOVA test. T0, n = 20; FU. n = 20. Figure S2: Differentially abundant genera in the urinary microbiome of StA arm patients with (RE) or without (NR) recurrence at different time points. A, Genera significantly more abundant in RE patients at T0 compared to NR patients at the same time point. B, Genera significantly more abundant in NR patients at follow-up (FU) compared to T0. Differential abundance analysis was performed using the MaAsLin2 package. The Benjamini–Hochberg procedure was applied to control the false discovery rate (FDR), and associations were considered significant at Q < 0.25. NR_T0, n = 13; NR_FU, n = 13; RE_T0, n = 7; RE_FU, n = 7.
