# Supplementary material for: Neoadjuvant Intravesical Mitomycin C for NMIBC: A Phase III Single-Center, Open-Label Randomized Clinical Trial
Source: Cancers (Basel). 2026 Apr 30;18(9):1444. doi: 10.3390/cancers18091444 (PMC13162961; doi:10.3390/cancers18091444)
Supplement: Supplementary file 1 [file cancers-18-01444-s001.zip › cancers-4175457-supplementary.pdf]

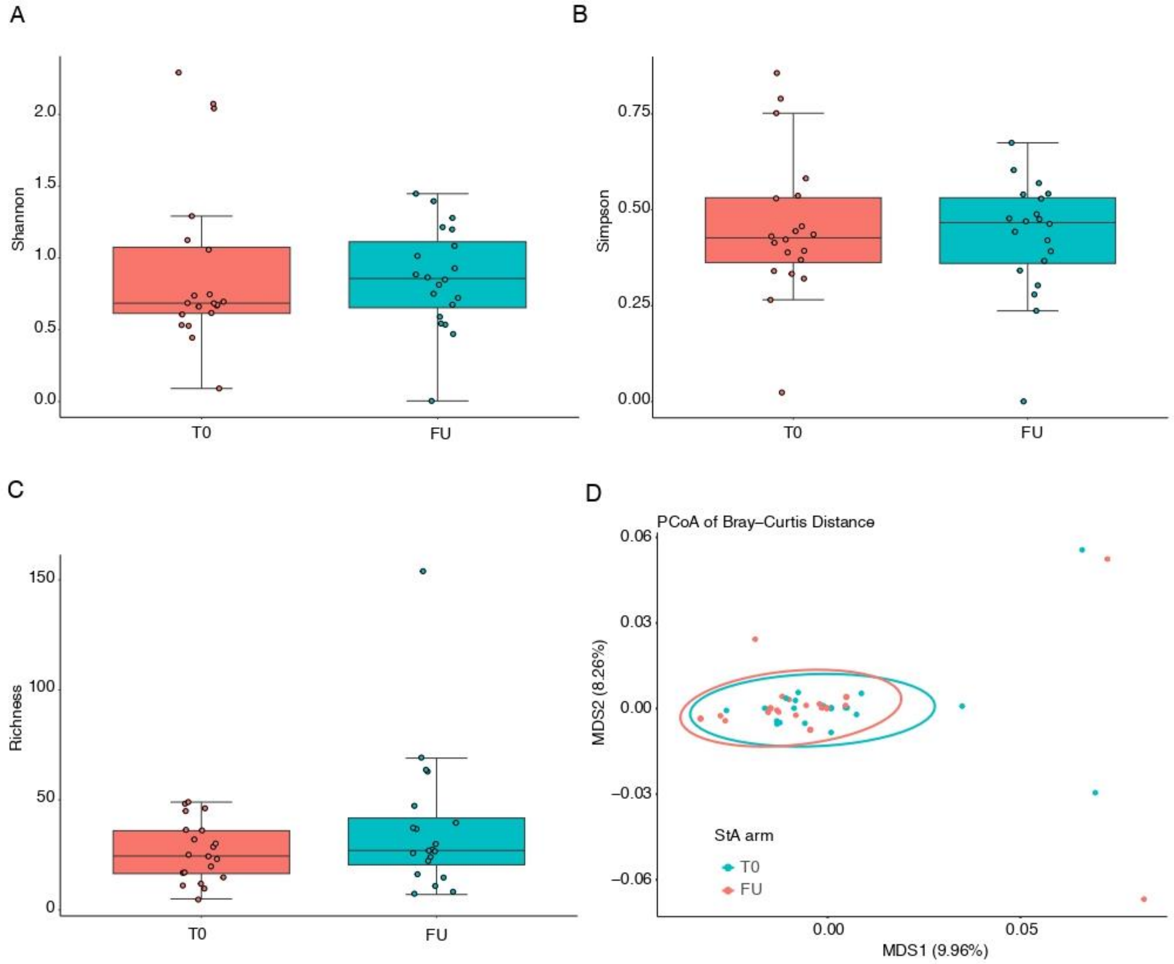

Figure S1. Alpha and Beta diversity analysis of the urinary microbiome in StA arm patients at T0 and at follow up (FU). A, Shannon index; B, Simpson index; C, Observed genera (Bacterial Richness). Statistical significance was assessed using a generalized linear model (GLM). D, Principal coordinate analysis (PCoA) of Bray Curtis distance of catheter-collected urine samples. Statistical significance was evaluated using the PERMANOVA test. T0, n=20; FU. n=20.

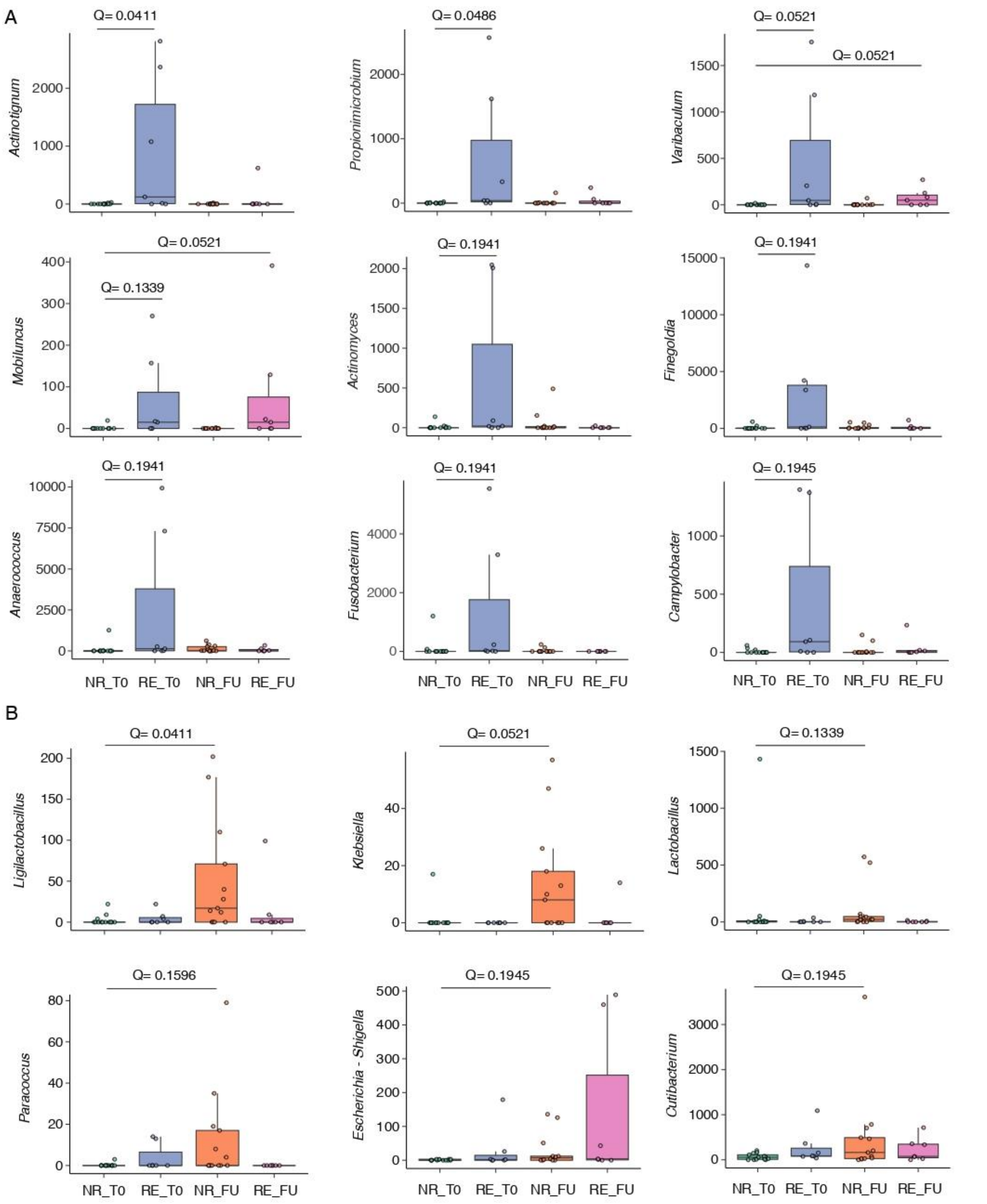

Figure S2. Differentially abundant genera in the urinary microbiome of StA arm patients with (RE) or without (NR) recurrence at different time points. A, Genera significantly more abundant in RE patients at T0 compared to NR patients at the same time point. B, Genera significantly more abundant in NR patients at follow-up (FU) compared to T0. Differential abundance analysis was performed using the MaAsLin2 package. The Benjamini–Hochberg procedure was applied to control the false discovery rate (FDR), and associations were considered significant at Q < 0.25. NR_T0, n=13; NR_FU, n=13; RE_T0 n=7; RE_FU, n=7.
